# Benefits of hypertonic saline for cardiothoracic organ transplantation with brain death donors

**DOI:** 10.1016/j.clinsp.2024.100530

**Published:** 2024-11-08

**Authors:** Cristiano de Jesus Correia, Ana Cristina Breithaupt-Faloppa, Luiz Felipe Pinho Moreira

**Affiliations:** Laboratório de Cirurgia Cardiovascular e Fisiopatologia da Circulação (LIM-11), Instituto do Coração (InCor), Faculdade de Medicina da Universidade de São Paulo (FMUSP), São Paulo, SP, Brazil

## Benefits of hypertonic saline for cardiothoracic organ transplantation

Organ transplantation remains the standard treatment for many end-stage organ diseases. Nevertheless, the number of solid organs available especially for heart and lung transplantations is far lower than the number of patients in the waiting lists.^1^ The main source for organ to transplantation continues to be brain-dead patients. However, brain death induces a series of responses including a massive release of catecholamines followed by hemodynamic instability, and hypoperfusion with microcirculatory impairment.^2^^,^^3^ Concomitantly, there is a progressive increase in inflammatory response with endothelial cell dysfunction and apoptosis, compromising the viability of the organs to be transplanted.^2^

The appropriate care of a potential organ donor represents the chance to increase the number of organs for transplantation. In this regard, the current guidelines in deceased organ donor management are based on a set of physiologic parameters with target values reflecting normal cardiovascular, respiratory, renal, and endocrine physiology. The maintenance of adequate intravascular volume associated with cardiac output augmentation has been recommended, if necessary, with the use of vasoactive medications. In parallel, a protective ventilatory strategy is advised with endocrine, electrolyte, and nutritional management. Besides, the consensus recommendations favor high-dose corticosteroid administration.^4^^,^^5^

For the management of the hemodynamic instability in brain-dead donors, isotonic crystalloid has been normally recommended to replace intravascular volume. However, fluid resuscitation in brain-dead donors is controversial, and inadequate fluid resuscitation leads to prolonged vasopressor use and inadequate oxygen delivery, resulting in organ ischemia and graft loss, especially in heart and lung transplantations.^6^ In this context, hypertonic saline is a known intravascular volume expander and some positive responses to its infusion are clearly related to brain death pathophysiology. It has been widely studied in several other pathologies, such as hemorrhagic shock, sepsis and ischemia and reperfusion injury, showing some advantages in relation to normal saline administration.^7^ The continuous hypertonic saline infusion also seems to reduce in-hospital mortality in brain-injured patients hospitalized in intensive care units.^8^

Sztark et al. (1995) were the first to use hypertonic saline in the treatment of potential donors in brain death. In that study, they showed that hypertonic saline can improve cardiac output and oxygen transportation.^9^ Nevertheless, this therapeutic option was never incorporated into the deceased organ donor management.

In recent studies, our group demonstrated that the use of hypertonic saline may reduce microcirculatory dysfunction and inflammation in experimental models of brain death, a situation that was also associated with improvement in cardiac performance and respiratory function. Besides its hemodynamic effects, hypertonic saline improved the density of perfused small vessels due to its effects on eNOS and the expression of the endothelin-1 protein, while reducing the inflammatory process by decreasing leukocyte adhesion and migration after the induction of brain death.^10^ The positive influence of hypertonic saline on microcirculatory changes has been extensively documented and also seems to be related to the hypertonicity, reducing the endothelial and red blood cell edema,^11^ and to the maintenance of coagulofibrinolytic homeostasis,^12^ whose dysregulation appears to be related to intravascular microthrombi formation observed after brain death.^13^

Regarding the cardiac effects, the observed data suggested that hypertonic saline ameliorates left ventricular systolic dysfunction and seems to reduce myocardial tissue compromise in brain-dead rats.^14^ Furthermore, hypertonic saline treatment induced the reversion of the altered expression of several miRNAs and the modulation of pathogenic pathways related to inflammatory process and to cellular apoptosis that was modified in the heart graft after brain death.^15^

The infusion of hypertonic saline also contributed to lung graft preservation after brain death induction. This positive influence was mainly related to the reduction of inflammatory cell infiltration into the lungs,^16^ while another study also demonstrated that hypertonic saline was effective in preventing the deterioration of elastic and resistive pulmonary components caused by brain death.^17^

In conclusion, the addition of the use of hypertonic saline in brain-dead organ donor management may have beneficial effects, especially for cardiothoracic organ transplantation. Given the absence of previous clinical data, randomized clinical studies are needed before developing recommendations for hypertonic saline use in the appropriate care of a potential organ donor.

## Declaration of competing interest

The authors declare no conflicts of interest.

## References

[1] (2023). Associação Brasileira de Transplante de Órgãos. Registro Brasileiro de Transplantes.

[2] Barklin A. (2009). Systemic inflammation in the brain-dead organ donor. Acta Anaesthesiol Scand.

[3] Simas R., Sannomiya P., Cruz J.W., Correia C.J., Zanoni F.L., Kase M. (2012). Paradoxical effects of brain death and associated trauma on rat mesenteric microcirculation: an intravital microscopic study. Clinics (Sao Paulo).

[4] Patel M.S., Abt P.L. (2019). Current practices in deceased organ donor management. Curr Opin Organ Transplant.

[5] Westphal G.A., Robinson C.C., Cavalcanti A.B., Gonçalves A.R.R., Guterres C.M., Teixeira C. (2021). Brazilian guidelines for the management of brain-dead potential organ donors. The task force of the Associação de Medicina Intensiva Brasileira, Associação Brasileira de Transplantes de Orgaos, Brazilian research in critical care network, and the general coordination of the national transplant system. Rev Bras Ter Intensiva.

[6] Korte C., Garber J.L., Descourouez J.L., Richards K.R. (2016). Hardinger K. Pharmacists’ guide to the management of organ donors after brain death. Am J Health-Syst Pharm.

[7] Pfortmueller C.A., Schefold J.C. (2017). Hypertonic saline in critical illness ‒ a systematic review. J Crit Care.

[8] Hourmant Y., Huard D., Demeure Dit Latte D., Bouras M., Asehnoune K., Pirrachio R. (2023). Effect of continuous infusion of hypertonic saline solution on survival of patients with brain injury: a systematic review and meta-analysis. Anaesth Crit Care Pain Med.

[9] Sztark F., Tentillier E., Thicoipé M., Gékière J.P., Lassié P., Petitjean M.E. (1995). Use of hypertonic saline solutions in the resuscitation of brain-dead organ donors. Transplant Proc.

[10] Correia C.J., Armstrong R., Carvalho P.O., Simas R., Sanchez D.C.J., Breithaupt-Faloppa A.C. (2019). Hypertonic saline solution reduces microcirculatory dysfunction and inflammation in a rat model of brain death. Shock.

[11] Cuschieri J., Gourlay D., Garcia I., Jelacic S., Maier R.V (2002). Hypertonic preconditioning inhibits macrophage responsiveness to endotoxin. J Immunol.

[12] Yang X., Chen Y., Li J., Chen L., Ren H., Liu Y. (2019). Hypertonic saline maintains coagulofibrinolytic homeostasis following moderate‑to‑severe traumatic brain injury by regulating monocyte phenotype via expression of lncRNAs. Mol Med Rep.

[13] Correia C.J., Ricardo da Silva F.Y., Armstrong R Junior, Vidal Dos Santos M., da Anunciação L.F., Sobral M.L.P. (2020). Sex differences in the coagulation process and microvascular perfusion induced by brain death in rats. Transpl Int.

[14] Magalhães D.M.S., Zanoni F.L., Correia C.J., Simas R., Soares R.G.F., Sannomiya P. (2019). Hypertonic saline modulates heart function and myocardial inflammatory alterations in brain-dead rats. J Surg Res.

[15] Ferreira L.R.P., Correia C.J., Zanoni F.L. (2022). Cardiac MicroRNA expression profile after experimental brain death is associated with myocardial dysfunction and can be modulated by hypertonic saline. Transplantation.

[16] Correia C.J., Coutinho E., Silva R.D.S., Soares R.G.F., Armstrong R., Ricardo-da-Silva F.Y. (2020). Hypertonic saline reduces cell infiltration into the lungs after brain death in rats. Pulm Pharmacol Ther.

[17] Ruiz L.M., de Oliveira Braga K.A., Nepomuceno N.A., Correia A.T., Ribeiro de Carvalho G.H., Vilela V.S. (2024). Effect of hypertonic saline solution on the ventilatory mechanics of lungs donated after brain death. J Surg Res.

